# Adherence to follow-up cervical cancer screening and its predictors among women living with HIV in Moshi municipality, Tanzania: a hospital-based retrospective cohort study (2019–2024)

**DOI:** 10.1136/bmjopen-2025-113748

**Published:** 2026-09-15

**Authors:** Dickson Doto, Laura J Shirima, Blandina Mmbaga, Deogratius Kinoko, Innocent B Mboya, Innocent H Peter Ughh, Alex Mremi, Patricia Swai, Bariki Mchome

**Affiliations:** 1 Department of Epidemiology and Biostatistics, School of Public Health, KCMC, Moshi Urban, Tanzania; 2 Kilimanjaro Clinical Research Institute (KCRI), Moshi, Tanzania; 3 School of Medicine, KCMC University, Moshi, Tanzania; 4 Department of Research and Programmes, Africa Academy for Public Health (AAPH), Dar es Salaam, Tanzania

**Keywords:** HIV & AIDS, Treatment Outcome, Mass Screening, Early Detection of Cancer, Cancer pain

## Abstract

**Abstract:**

**Objective:**

The effectiveness of cervical cancer screening as a preventive strategy for cervical cancer substantially depends on timely and consistent adherence to recommended follow-up screenings. This study aimed to determine adherence to the recommended follow-up cervical cancer screening, characterise the timing of adherence and determine its associated predictors among women living with HIV (WLHIV) who undertook their first screening between 2019 and 2021 in Moshi Municipality, northern Tanzania.

**Design:**

We conducted a retrospective cohort study between 2019 and 2021.

**Setting:**

Healthcare facilities in Moshi municipality, Kilimanjaro region, northern Tanzania.

**Participants:**

2030 WLHIV who undertook their first screening in healthcare facilities in Moshi municipality between 2019 and 2021.

**Primary outcome measures:**

Adherence to a follow-up cervical cancer screening, defined as the subsequent screening test conducted to detect precancerous development in the cervix carried out after the first screening. For the sub-analysis assessing the timing of adherence to a follow-up screening, this outcome was further categorised into four distinct categories: early adherence, good adherence, poor adherence, extremely poor adherence based on the time elapsed between the first screening and subsequent follow-up screening. Predictors of adherence were assessed using multivariable Poisson regression models with robust SEs and reported as adjusted risk ratios (ARR) with 95% CIs.

**Results:**

Among 2030 WLHIV who undertook the first screening, only 33.2% adhered to a follow-up screening. Among these, only 21.4% (95% CI 18.4% to 24.7%) demonstrated good adherence, meaning they attended a follow-up within a month after the scheduled date. Also, adherence was higher in private healthcare facilities (ARR=1.57; 95% CI 1.35 to 1.82), lower for WLHIV who had their first screening at outreach facilities (ARR=0.28; 95% CI 0.19 to 0.39) and higher for those who were diagnosed with an abnormal screening outcome in their first screening (ARR=2.62; 95% CI 2.26 to 3.05). Adherence also increased with age and parity.

**Conclusion:**

Adherence to follow-up cervical cancer screening among WLHIV in Moshi municipality was suboptimal, with only one-third of WLHIV adhering to these screenings. Strengthening recall systems particularly in public and outreach facilities and implementing targeted interventions for younger WLHIV, those with no or fewer children and those who had normal screening outcomes in the first screening is essential to improve adherence to recommended follow-up screening.

**Strengths and limitations of this study**

- The study involved a large cohort of study participants, who were followed up for an extended period of time.
- The dataset lacked key socio-demographic variables, thus limiting the assessment of social determinants of adherence.
- Follow-up of study participants was only tracked at healthcare facilities where they undertook their first screening, this potentially underestimated true adherence.

## Introduction

 Cervical cancer screening is a crucial strategy in the fight against cervical cancer because it enables the early detection and treatment of precancerous lesions before they progress to invasive cervical cancer.^1 2^ Early cervical cancer screening combined with timely follow-up screenings can significantly reduce the burden of the disease.^2 3^ Studies have demonstrated that consistent adherence to annual follow-up screening can reduce the burden of the disease by up to 90%.^4^ Much of the global disease burden is borne in sub-Saharan Africa (SSA), where approximately 85% of cervical cancer cases and 90% of related deaths occur.^5^ This is partly due to the confounding effect of high prevalence of HIV/AIDS in the region, which predisposes women living with HIV (WLHIV) to a higher susceptibility of developing invasive cancer than women living without HIV.^6 7^ Moreover, adherence to follow-up cervical cancer screening in other SSA settings remains suboptimal. Reports from Nigeria and Botswana indicate that only 37.8% and 40.1% respectively adhered to a follow-up screening after they undertook their first screening.^8 9^

In Tanzania, the minimum eligible age for cervical cancer screening among WLHIV has been lowered to 25 years, given their increased risk of developing invasive cervical cancer, compared with 30 years required for women in the general population.^10^ WLHIV are recommended to undertake an annual follow-up screening compared with 3 years recommended for those in the general population.^11^

In Tanzania, it was reported in 2024 that 36.7% of eligible WLHIV had already undertaken their first cervical cancer screening by 2022.^12^ To date, no further studies have reported on the proportion of WLHIV who later adhered to a follow-up screening. Also, several predictors including age, parity, marital status and screening outcomes in the first screening have been reported to be predictors of adherence to a follow-up screening. However, these findings have been inconsistent across the globe, and no study conducted in the country or similar settings has conclusively determined those predictors.^13–15^ This study aimed to determine adherence to the recommended follow-up cervical cancer screening, characterise the timing of adherence, and determine its associated predictors among WLHIV who undertook their first screening between 2019 and 2021 in Moshi Municipality, northern Tanzania.

## Methodology

### Data source

Cervical cancer screening data were obtained from the National Health Management Information System (HMIS), locally known in Swahili language as *Mfumo wa Taarifa za Uendeshaji wa Huduma za Afya*. This system comprises both electronic registries - which contain aggregated monthly summaries of screening data and hardcopy registries - which include detailed individual-level screening data. In addition, participants’ HIV-related data were extracted from the Care and Treatment Clinic (CTC2) database. Participant names and/or unique identification numbers available at each healthcare facility were used to manually link the HMIS and CTC2 databases, thereby enabling the retrieval of individual-level HIV-related data from CTC2 one by one.

### Study design and setting

**Figure 1:**
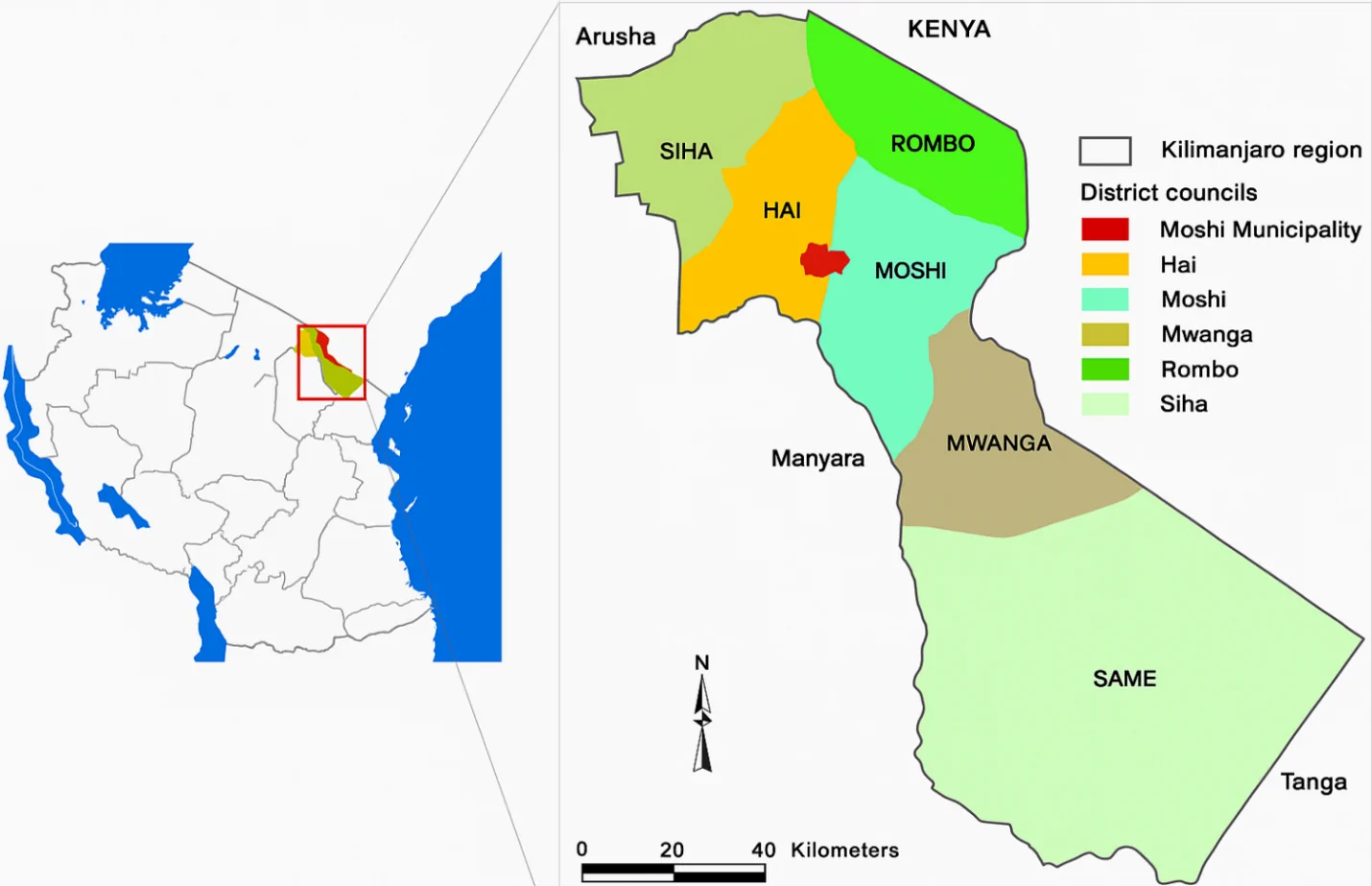
Map of Moshi Municipality (Moshi Urban).

We conducted a hospital-based retrospective cohort study in healthcare facilities that provide cervical cancer screening services in Moshi Municipality, northern Tanzania. Four of the six eligible facilities were purposively selected. This included Mawenzi Regional Referral Hospital, St. Joseph Hospital, Majengo Health Centre and *Chama cha Uzazi na Malezi Bora* (UMATI). Facility selection ensured representation across different levels of healthcare delivery, that is, a dispensary, a healthcare centre, a district hospital and a regional referral hospital. Additionally, it also aimed to achieve a balanced inclusion of both public and private healthcare facilities in the study (figure 1).

### Study participants

**Figure 2:**
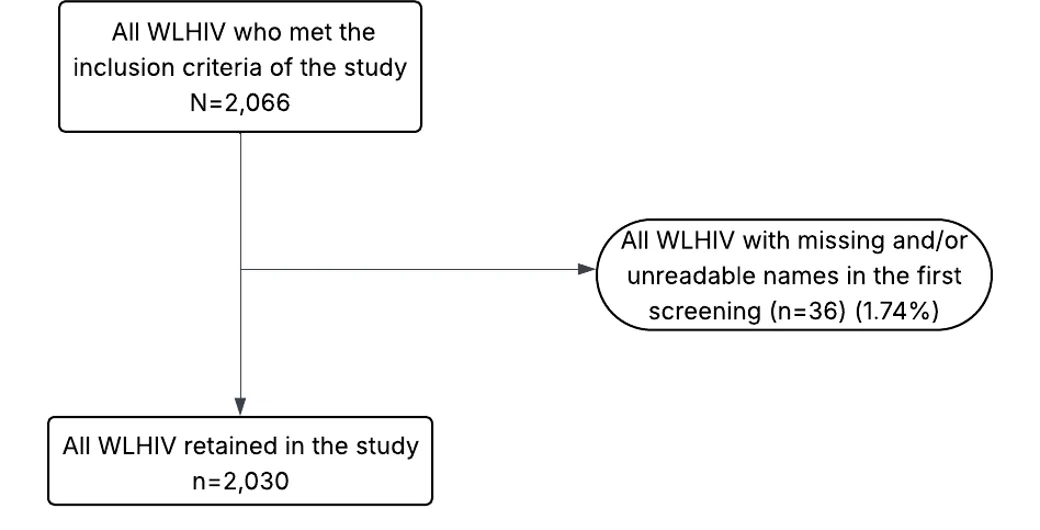
Flowchart diagram for the selection of the study participants. WLHIV, women living with HIV.

The study enrolled 2066 WLHIV who undertook their first cervical cancer screening at the selected healthcare facilities in the Municipality from 2019 to 2021 and followed through 2024. A total of 36 (1.74%) participants were excluded because their records contained missing and/or unreadable names, which made it impossible to track or link them to their follow-up screening. The final analytic sample included 2030 WLHIV (figure 2).

### Variables

The outcome variable in this study was adherence to a follow-up cervical cancer screening. This was defined as the subsequent screening test conducted to detect precancerous development in the cervix carried out after the first screening. According to Tanzania cervical cancer screening service delivery guidelines, this test is recommended to be undertaken a year (12 months) after the first screening.^16^ It was a binary variable - categorised as “adhered” and “not adhered”.

Furthermore, for the sub-analysis aiming to assess the timing of adherence, the outcome variable was further classified into several distinct categories based on national service delivery guidelines and evidence from previous studies: This included early adherence – defined as compliance with the scheduled follow-up screening at any time period prior to the scheduled date; good adherence – defined as compliance with scheduled follow-up screening within 1 month after the scheduled date; poor adherence – defined as adherence to a follow-up screening between 1 month and less than a year after the scheduled date; and extremely poor adherence – defined as adherence to a follow-up screening attended at least a year after the scheduled follow-up screening.^11 17^

The exposure variables included socio-demographic and clinical characteristics of WLHIV. The socio-demographic variables included the name of the patient (only for data linkage), area of residence (Kilimanjaro region, other regions), age (<25 years, 25–34 years, 35–44 years, 45–54 years and ≥55 years) and parity (0–1 child, 2–4 children and ≥5 children).

**Figure 3:**
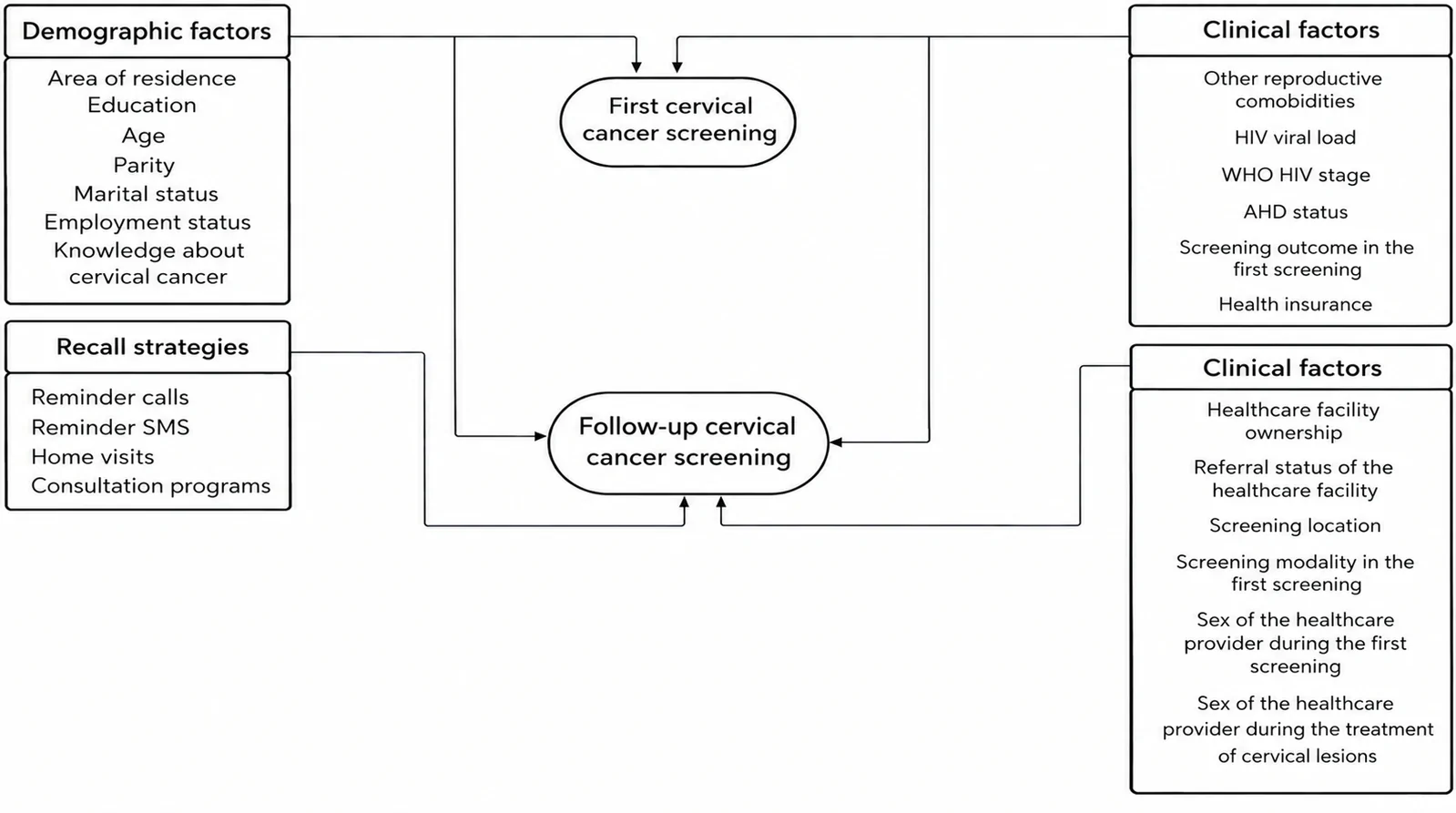
Conceptual framework for follow-up cervical cancer screening among WLHIV. AHD, Advanced HIV Disease.

The clinical variables included the screening location (health facility, outreach facility), referral status of the healthcare facility (non-referral facility, referral facility), presence of other comorbidities (yes, no), WHO HIV stage of the patients (stage I, stage II, stage III, stage IV), HIV viral load of the patients at the first screening (Undetected, Suppressed, Unsuppressed), screening outcome in the first screening (normal, abnormal), screening modality at the first screening (Visual Inspection with Acetic acid (VIA), other), sex of the healthcare provider during the first screening and during the treatment of the precancerous lesions (male, female), treatment modality of the precancerous lesions (cryotherapy, thermocoagulation, LEEP, other), size of the precancerous lesions at the first screening (small, large), suspected to have already developed the invasive cervical cancer during the first screening (yes, no), Advanced HIV Disease (AHD) development status during the first screening (yes, no), information source for HIV positive status of the patient (self-declared, CTC2 card, PICT, RCH4 card, RCH5 card, VCT card), qualification of the healthcare provider in the first screening and during the treatment of precancerous lesions (nurse, assistant medical officer, medical officer, medical specialist, other) and patient CTC2 number (figure 3).

### Statistical analysis

Descriptive statistics were used to summarise the socio-demographic and clinical characteristics of the study participants. Categorical variables were summarised using frequencies and proportions, and continuous variables using means and SD. The differences in the proportion of adherence to a follow-up cervical cancer screening by independent variables were assessed using the χ^2^ test for proportions. Multivariable Poisson regression models with robust SEs were used to estimate the adjusted risk ratio (ARR) and their corresponding 95% CIs for the predictors of adherence to a follow-up cervical cancer screening.

Before model fitting, the assumptions for the Poisson regression model were carefully examined. Outliers in the continuous variables – age and parity were identified by plotting boxplot diagrams. Given the presence of extreme values for age and parity variables, the winsorisation technique was performed to remove their influence while preserving the overall data structure.^17^ The new refined versions of those variables without outliers were created and then categorised according to established evidence from previous studies and incorporated into the multivariable model. Spearman correlation analysis was conducted to assess the presence of multicollinearity among the independent variables. Evidence of multicollinearity was observed among healthcare facility ownership, referral status and treatment modality for precancerous lesions (correlation coefficient, *r*=0.70). To remove the effects of multicollinearity, referral status and treatment modality for precancerous lesions were excluded from the multivariable analyses.

Several HIV-related variables in the original dataset had substantial levels of missing observations. The level of missingness was 30.1% for WHO HIV stage (categorical variable), 27.3% for AHD development status (categorical variable) and 18.9% for HIV viral load (continuous variable). Such levels of missingness pose a great risk of reducing statistical power, introducing biased parameter estimates and potentially affecting the validity of the study findings if not properly addressed. Therefore, a sensitivity analysis was conducted by comparing the regression results from models that included these variables with missing observations and models with these variables in which the missing observations had been addressed using the mode imputation method. Significant differences were observed between the model estimates, suggesting that the missing data could greatly influence the study findings. Consequently, the versions of these variables with missing observations addressed by the mode imputation method were retained and included in the final regression analyses.

Moreover, variables with a p value <0.25 in the crude analysis, those deemed to have potential confounding effects and variables with established epidemiological relevance to follow-up cervical cancer screening were considered for inclusion in the multivariable model. The final model was selected using the Akaike Information Criterion (AIC), with lower AIC values indicating a better model fit. The final multivariable Poisson regression model with robust SEs included the following variables: age at the first screening, parity at the first screening, presence of other reproductive comorbidities at the first screening, healthcare ownership, screening location, screening outcomes in the first screening, HIV viral load, size of the cervical precancerous lesions, area of residence and suspected of developing invasive cervical cancer at the first screening, AHD development status and WHO HIV stage in the first screening.

## Results

### Participant characteristics

A total of 3020 WLHIV who undertook their first cervical cancer screening between 2019 and 2021 were included in the study. Their mean age was 39.6 years (SD 9.4). 36.5% were aged 35–44 years, and nearly all (99.6%) resided in Kilimanjaro region. Of all WLHIV, the majority, 85% undertook their first screening at a healthcare facility rather than an outreach facility. Also, their median parity was 3, and the majority, 71.8% had 2–4 children. Also, of all WLHIV, 11.6% had other reproductive comorbidities including cervicitis, pelvic inflammatory disease (PID), uterine fibroid, vaginal candidiasis and vaginal discharge. Moreover, 29.1% were classified as having WHO HIV stage I, the majority 72.7% had undetectable HIV levels (<50 copies/mm^3^) and the majority 66.4% had not yet developed AHD. In addition, all of the screening procedures were performed by the VIA method and all healthcare providers involved in the screening procedures were females (table 1).

**Table 1:** Socio-demographic and clinical characteristics of the study participants (n=2030)

Characteristics	n (%)
Age (years)	
Mean (SD)	39.6 (9.4)
<25	113 (5.6)
25–34	509 (25.0)
35–44	741 (36.5)
45–54	592 (29.2)
≥55	75 (3.7)
Area of residence	
Kilimanjaro region	2022 (99.6)
Other regions	8 (0.4)
Screening location	
Healthcare facility	1725 (85.0)
Outreach facility	305 (15.0)
Healthcare facility involved	
Majengo Health Centre	712 (35.1)
Mawenzi Regional Referral Hospital	1080 (53.2)
St. Joseph Hospital	198 (9.8)
UMATI	40 (1.9)
Parity at the first screening	
Mean (SD)	2.7 (1.4)
0–1	374 (18.4)
2–4	1458 (71.8)
≥5	198 (9.8)
Information source for HIV status of the patients	
Self-declared	155 (7.6)
CTC card	1714 (84.4)
PICT	157 (7.7)
RCH4 card	3 (0.2)
RCH5 card	1 (0.05)
Presence of other reproductive comorbidities at the first screening	
No	1794 (88.4)
Yes	236 (11.6)
WHO HIV stage of the patients at the first screening*	
I	590 (29.1)
II	273 (13.5)
III	341 (16.8)
IV	87 (4.3)
HIV viral load of the patients at the first screening (copies/mL)*	
Undetected (<50)	1475 (72.7)
Suppressed (50–1000)	62 (3.1)
Unsuppressed (>1000)	31 (1.5)
Advanced HIV Disease (AHD) status of the patients at the first screening*	
No	1347 (66.4)
Yes	20 (1.0)

* n (%) do not tally to 100% due to missing values.

AHD, Advanced HIV Disease; CTC card, Care and Treatment Clinic card for people living with HIV; PICT, Provider-Initiated HIV Counselling and Testing card; RCH4 card, Reproductive and Child Health Card for Pregnant Women; RCH5 card, Reproductive and Child Health Card for Family Planning Services; UMATI, Chama cha Uzazi na Malezi Bora.

### Adherence to follow-up cervical cancer screening by WLHIV characteristics

Among 2030 WLHIV who were enrolled in the study, 673 (33.2%) (95% CI 31.1% to 34.8%) adhered to a follow-up screening. Among them, only 144 (21.4%) (95% CI 18.4% to 24.7%) demonstrated good adherence, meaning they attended a follow-up screening within 1 month after the scheduled date. Others 177 (26.3%) (95% CI 23.0% to 29.8%) demonstrated early adherence, meaning they attended a scheduled follow-up screening at any time period prior to the scheduled date, 136 (20.2%) (95% CI 17.2% to 23.4%) demonstrated poor adherence, meaning they attended a follow-up screening between 1 month and less than a year after the scheduled date whereas 216 (32.1%) (95% CI 28.6% to 35.8%) demonstrated extremely poor adherence, meaning they attended a follow-up screening at least a year after the scheduled date.

Moreover, among WLHIV who adhered to a follow-up screening, 32.2% were residing in Kilimanjaro region, and 37.3% undertook their first screening at the healthcare facility rather than an outreach facility and a large proportion, 41.3%, of the healthcare facilities they screened at were non-referral healthcare facilities. Also, 35.1% were aged 35–44 years, 33.8% had 2–4 children at the time of their first screening and 39.5% were in WHO HIV stage I at the time of their first screening. Also, 37.1% had undetected viral load levels (<50 copies/mL) at the time of their first screening and 37.9% had not yet developed AHD at the time of their first screening. Also, 31.0% of WLHIV were diagnosed with normal screening outcomes (cervical precancerous lesions) at their first screening, and 90.2% of those diagnosed with abnormal screening outcomes were treated with a thermocoagulation procedure, and 33.1% of the diagnosed lesions were categorised as small lesions. In addition, 32.8% of those WLHIV had not yet developed other reproductive comorbidities at the time of their first screening (table 2).

**Table 2:** Adherence to a follow-up cervical cancer screening by socio-demographic and clinical factors among the study participants (n=2030)

Variable	Adherence status		P value
	Adhered n (%)	Not adhered n (%)	
Area of residence			0.624
Kilimanjaro region	671 (33.2)	1351 (66.8)	
Other regions	2 (25.0)	6 (75.0)	
Screening location			<0.0001*
Healthcare facility	644 (37.3)	1081 (62.7)	
Outreach facility	29 (9.5)	276 (90.5)	
Referral status of the healthcare facility			<0.0001*
Non-referral facility	392 (41.3)	558 (58.7)	
Referral facility	281 (26.0)	799 (74.0)	
Age (years)			**0.004**
<25	20 (17.7)	93 (82.3)	
25–34	160 (31.4)	349 (68.6)	
35–44	260 (35.1)	481 (64.9)	
45–54	205 (34.6)	387 (65.4)	
≥55	28 (37.3)	47 (62.7)	
Parity at the first screening			<0.0001*
0–1	92 (24.6)	282 (75.4)	
2–4	493 (33.8)	965 (66.2)	
≥5	88 (44.4)	110 (55.6)	
Presence of other reproductive comorbidities at the first screening			0.320
No	588 (32.8)	1206 (67.2)	
Yes	85 (36.0)	151 (64.0)	
WHO HIV stage of the patients at the first screening			0.625
I	233 (39.5)	357 (60.5)	
II	100 (36.6)	173 (63.4)	
III	128 (37.5)	213 (62.5)	
IV	38 (43.7)	49 (56.3)	
HIV viral load of the patients at the first screening (copies/mL)			0.949
Undetected (*<*50)	548 (37.1)	927 (62.9)	
Suppressed (50–1000)	22 (35.5)	40 (64.5)	
Unsuppressed (*>*1000)	12 (38.7)	19 (61.3)	
Screening outcome in the first screening			<0.0001*
Normal	601 (31.0)	1335 (69.0)	
Abnormal	72 (76.6)	22 (23.4)	
Treatment modality of the precancerous lesions in the first screening			**0.003**
Cryotherapy	17 (60.7)	11 (39.3)	
Thermocoagulation	46 (90.2)	5 (9.8)	
Size of the cervical precancerous lesion at the first screening			0.257
Small	671 (33.1)	1356 (66.9)	
Large	2 (66.7)	1 (33.3)	
Suspected of developing invasive cervical cancer at the first screening			**0.019**
No	667 (33.0)	1355 (67.0)	
Yes	6 (75.0)	2 (25.0)	
AHD development status among the patients			0.110
No	510 (37.9)	837 (62.1)	
Yes	4 (20.0)	16 (80.0)	

Bolded p-values: Statistically significant p-values

AHD, Advanced HIV Disease.

### Predictors of adherence to follow-up cervical cancer screening among WLHIV

After adjusting for the effect of other variables in the multivariable model, we found that WLHIV who had their first screening at the private facility were 1.57 times more likely to adhere to a follow-up screening compared with those who screened at a public facility (ARR=1.57, 95% CI 1.35 to 1.82). WLHIV who had their first screening at an outreach facility were 0.28 times less likely to adhere to a follow-up screening compared with those who went to a regular healthcare facility (ARR=0.28, 95% CI 0.19 to 0.39). Also, WLHIV who were diagnosed with abnormal cervical cancer screening outcomes in their first screening were 2.62 times more likely to adhere to a follow-up screening compared with those diagnosed with normal screening outcomes (ARR=2.62, 95% CI 2.26 to 3.05). WLHIV who had not already developed AHD at the time of the first screening were 0.44 times less likely to adhere to a follow-up screening compared with those who had already developed AHD at the time of the first screening (ARR=0.44, 95% CI 0.22 to 0.92). In addition, adherence to a follow-up screening was found to increase with age (ie, ≥55 years, ARR=1.48, 95% CI 1.07 to 2.44) and parity (ie, ≥5 children, ARR=1.62, 95% CI 1.27 to 2.07) (table 3).

**Table 3:** Multivariable Poisson regression analysis for predictors of follow-up cervical cancer screening among WLHIV who undertook their first screening between 2019 and 2021 in Moshi Municipality (n=2030)

Variable	CRR (95% CI)	P value	ARR (95% CI)	P value
Area of residence				
Kilimanjaro region	1.00		1.00	
Other regions	0.75 (0.23 to 2.51)	0.644	0.61 (0.22 to 1.65)	0.328
Healthcare facility ownership				
Public	1.00		1.00	
Private	1.77 (1.54 to 2.03)	<0.001*	1.57 (1.35 to 1.82)	<0.001*
Screening location				
Health facility	1.00		1.00	
Outreach facility	0.25 (0.17 to 0.36)	<0.001*	0.28 (0.19 to 0.39)	<0.001*
Age (years)				
<25	1.00		1.00	
25–34	1.85 (1.20 to 2.85)	**0.005**	1.64 (1.08 to 2.48)	**0.019**
35–44	2.07 (1.36 to 3.16)	**0.001**	1.62 (1.07 to 2.46)	**0.022**
45–54	2.04 (1.33 to 3.12)	**0.001**	1.52 (1.09 to 2.32)	**0.042**
≥55	2.2 (1.32 to 3.64)	**0.002**	1.48 (1.07 to 2.44)	**0.047**
Parity at the first screening				
0–1	1.00		1.00	
2–4	1.37 (1.13 to 1.66)	**0.001**	1.28 (1.05 to 1.55)	**0.011**
≥5	1.81 (1.42 to 2.29)	<0.001*	1.62 (1.27 to 2.07)	<0.001*
Presence of other reproductive comorbidities at the first screening				
No	1.00		1.00	
Yes	1.09 (0.92 to 1.32)	0.311	1.03 (0.85 to 1.23)	0.789
WHO HIV stage of the patients at the first screening				
I	1.00		1.00	
II	1.19 (1.0 to 1.42)	**0.046**	1.16 (0.98 to 1.37)	0.075
III	1.22 (1.05 to 1.44)	**0.012**	1.08 (0.98 to 1.37)	0.294
IV	1.42 (1.05 to 1.44)	**0.006**	1.14 (0.89 to 1.47)	0.277
HIV viral load of the patients at the first screening (copies/mL)				
Undetected (*<*50)	1.00		1.00	
Suppressed (50–1000)	1.07 (0.76 to 1.51)	0.676	1.16 (0.85 to 1.60)	0.347
Unsuppressed (*>*1000)	1.17 (0.75 to 1.84)	0.484	1.47 (0.93 to 2.33)	0.096
Screening outcome in the first screening				
Normal	1.00		1.00	
Abnormal	2.46 (2.17 to 2.81)	**<0.001**	2.62 (2.26 to 3.05)	<0.001*
Size of the cervical precancerous lesion at the first screening				
Small	1.00		1.00	
Large	2.01 (0.90 to 4.49)	0.087	0.79 (0.34 to 1.86)	0.595
Suspected of developing invasive cervical cancer at the first screening				
No	1.00		1.00	
Yes	2.27 (1.52 to 3.41)	<0.001*	0.93 (0.61 to 1.39)	0.713
Advanced HIV Disease (AHD) development status				
No	1.00		1.00	
Yes	0.60 (0.25 to 1.45)	0.256	0.44 (0.22 to 0.92)	**0.028**

ARR, Adjusted Risk Ratio; CRR, Crude Risk Ratio.

## Discussion

This study found that only 33.2% of WLHIV who undertook their first cervical cancer screening in the selected healthcare facilities had adhered to a follow-up screening. This suboptimal adherence could have been attributed to one or a combination of several factors, including transportation challenges, long distance to the healthcare facilities, fear of HIV stigma, inadequate knowledge among WLHIV about the importance of adhering to such screenings and inadequate patient recall systems at the facility level as it has been reported in other similar studies.^17 18^ Nevertheless, this finding is consistent with a similar study conducted in Nigeria, which reported a suboptimal adherence of 37.8% to a follow-up screening.^18^ Such consistency suggests that the challenges of adherence to a follow-up cervical cancer screening among WLHIV may be common in low-resource settings.

Among WLHIV who adhered to a follow-up screening, only 21.4% demonstrated good adherence, defined as attending the scheduled follow-up visit within the recommended timeframe according to the national cervical cancer screening service delivery guidelines.^18 19^ This indicates that the majority did not comply within the recommended period, instead they attended either earlier or later than the scheduled follow-up date. This finding highlights potential gaps in cervical cancer screening service delivery, particularly regarding WLHIV’s understanding of the recommended timing and importance of follow-up screening. This also suggests possible deficiencies in how healthcare providers communicate follow-up screening instructions and timelines to the screened WLHIV, regardless of their first screening outcomes, as stipulated in the national guidelines. Timely adherence to a follow-up screening is especially critical in cases where abnormal screening outcomes in the first screening have been misclassified as normal, or where treatment of precancerous lesions was incomplete or unsuccessful. A delayed follow-up screening in such scenarios may lead to detection of such abnormal screening outcomes at a point where precancerous lesions have already progressed into invasive cervical cancer – an outcome which is associated with more complex treatment options and reduced survival chances. Nevertheless, this finding is also consistent with evidence from Denmark, where only 26% of women demonstrated good (recommended) adherence to follow-up screening after their first screening, suggesting that challenges with timely follow-up screenings are not limited to low-resource settings but may also occur in high-income contexts.^17^

The study also highlighted a strong association between the ownership of healthcare facilities and adherence to a follow-up screening among WLHIV. This finding underscores the existing disparities between private and public healthcare facilities in promoting and facilitating follow-up care. Specifically, WLHIV who received their first screening from private healthcare facilities were more likely to adhere to a follow-up screening compared to those who went to public healthcare facilities. This finding may be attributed to the generally higher quality of service delivery, better provider–client relationships and greater patient satisfaction often reported in private healthcare facilities in Tanzania and other settings in SSA. Such factors may encourage WLHIV to return for follow-up screening in private healthcare facilities as compared with public healthcare facilities.^20 21^ In addition, understaffing, which remains a common challenge in public healthcare facilities, may contribute to long queues and extended waiting times, particularly for Reproductive, Maternal, Newborn, Child and Adolescent Health (RMNCAH) and HIV-related services, thereby discouraging women from returning for follow-up screening due to such previous bad experiences with service delivery.^22^ These disparities highlight the need for targeted strategies aimed at strengthening follow-up screening services within public healthcare settings, including increasing healthcare workforce capacity in RMNCAH services which will then help to reduce waiting times and enhance patient satisfaction through improved provider–client relationships and communication.

The study also revealed a strong association between the location where the first cervical cancer screening took place and adherence to a follow-up screening among WLHIV. Specifically, WLHIV who undertook their first cervical cancer screening at the regular healthcare facility were significantly more likely to adhere to a follow-up screening compared with those who had their first screening at outreach facilities. This finding suggests that healthcare facility-based screenings may be offering more structured follow-up mechanisms, including proper documentation, better follow-up appointment scheduling and more effective communication with patients. In contrast, outreach settings, while essential for expanding accessibility to screening services, may face challenges in maintaining patient records, providing timely reminders and ensuring continuity of care.^23^ These results highlight the importance of integrating strong follow-up mechanisms into outreach programmes or linking them effectively with fixed healthcare facilities to improve adherence to follow-up screening among WLHIV.^24^

The study also revealed a significant association between the age of the participants and adherence to follow-up screening among WLHIV. Specifically, the findings have shown that older WLHIV were more likely to adhere to a follow-up screening compared with their younger counterparts.^25^ This trend may reflect increased health awareness, risk perception, health-seeking behaviour and frequent interaction with healthcare services among older WLHIV.^26^ Conversely, younger WLHIV may be experiencing barriers such as limited health knowledge, competing priorities or lower perceived susceptibility to cervical cancer. This finding suggests the need for age-targeted health education and counselling strategies, particularly focusing on younger WLHIV, aiming to improve their adherence to a follow-up screening. Nevertheless, this finding is consistent with previous studies conducted in Ethiopia and Ivory Coast, which similarly reported higher adherence to follow-up cervical cancer screening among older WLHIV.^27 28^

The study also found a statistically significant association between parity and adherence to follow-up screening among WLHIV. Specifically, the more children WLHIV had, the more likely they were to adhere to a follow-up screening compared with those with fewer or no children.^29^ The observed association may be explained by the fact that WLHIV with multiple births tend to have more frequent contact with maternal and child health services, which may enhance their exposure to health education, screening opportunities and the overall importance of preventive healthcare. Also, having more children may reflect greater life experience and a heightened awareness of reproductive health risks, including cervical cancer, and thus encourage better health-seeking behaviour.^30^ This finding highlights the importance of integrating cervical cancer education and screening promotion into maternal health services, especially for women with no or fewer children who may not routinely engage with healthcare systems. Also, this finding is consistent with the findings from similar studies conducted in Ethiopia, Uganda and Zimbabwe.^27 29 31^

The study also revealed a strong association between the screening outcome in the first cervical cancer screening and subsequent adherence to a follow-up screening among WLHIV. Specifically, WLHIV who were diagnosed with abnormal screening outcome in their first screening were more likely to adhere to a follow-up screening compared with those who were diagnosed with a normal first screening outcome. This trend may have been attributed to increased concern of perceived vulnerability among WLHIV with abnormal findings, which may prompt them to take recommended follow-up actions more seriously.^26^ Additionally, the emotional impact of an abnormal screening outcome may also enhance motivation to engage with healthcare services, follow-up treatment protocols, and attend follow-up visits. In contrast, WLHIV with a normal screening outcome might perceive themselves as being at low risk and therefore may not prioritise returning for a follow-up screening.^32^ This finding emphasises the need for tailored counselling for WLHIV who undertook their first screening, regardless of their screening outcomes in their first screening. This will potentially help to ensure all WLHIV who undertook their first screening understand the importance of adhering to a follow-up screening. In addition, this finding was also consistent with the findings from a similar study conducted in Zimbabwe.^33^

The study also found a strong association between AHD development status and adherence to a follow-up screening among WLHIV. Specifically, WLHIV who had not developed AHD were more likely to adhere to a follow-up screening compared with those who had developed AHD at the time of their first screening. This pattern may be due to the fact that WLHIV who have not developed AHD are generally healthier and likely able to attend scheduled appointments compared with those with AHD who may be facing additional health burdens, reduced morbidity or competing clinical priorities that hinder their ability to adhere to scheduled follow-up screening.^33^ This finding highlights the need for targeted support strategies to improve follow-up adherence among WLHIV with AHD. Nonetheless, this finding should be interpreted cautiously due to the small number of WLHIV who have developed AHD in the study and had adhered to follow-up screening, which may have compromised the validity and reliability of the given estimates. Therefore, although this finding is statistically significant, it may not be sufficient to provide evidence to inform policy or programmatic decisions unless confirmed in studies with a larger number of WLHIV who have developed AHD.

### Strength and limitations of the study

To our knowledge, our study is the first in the country and other SSA countries to examine follow-up cervical cancer screening among WLHIV and classify the timing of adherence. The study also included a large cohort followed over an extended period, allowing for a comprehensive and reliable assessment of factors associated with adherence. Moreover, the original data source for this study lacked key socio-demographic variables including education level, marital status and employment status, thus limiting a more in-depth analysis of social determinants which the potential of predicting adherence to a follow-up screening. In addition, follow-up was only tracked within the healthcare facilities where the first screening was conducted. Women who attended follow-up screening elsewhere may have been misclassified as non-adherent, potentially leading to an underestimation of true adherence levels.

## Conclusion

Adherence to a follow-up cervical cancer screening among WLHIV was suboptimal, with only a third adhering to it. Among them, an even smaller proportion did so within the recommended timeframe. This highlights a critical gap in the continuity of the cervical cancer screening programme among WLHIV in the country. Also, predictors significantly associated with adherence to a follow-up screening among WLHIV included healthcare facility ownership, screening location, age, parity, AHD development status and screening outcome in the first screening.

Healthcare providers, particularly in public healthcare facilities, in collaboration with other stakeholders, should implement targeted strategies aimed at improving patient satisfaction and strengthening provider-client relationships by fostering rapport building, effective communication and patient-centred care throughout the cervical cancer screening process. These strategies may include ensuring implementation of respectful and compassionate care, improving counselling practices, enhancing privacy and confidentiality during service provision and creating supportive healthcare environments that encourage WLHIV to freely engage with healthcare providers and adhere to scheduled follow-up screenings. Furthermore, the Ministry of Health should ensure that standardised counselling and health education is provided in all healthcare facilities which provide cervical cancer screening to ensure that all WLHIV who undertook their first screening are adequately informed about the importance of timely follow-up screening and the risks associated with delayed adherence.

Additionally, stakeholders in the health sector should collaborate with facility-level clinicians to plan and implement age- and parity-targeted interventions aiming to specifically promote follow-up screening among younger WLHIV and/or those with fewer or no children who had remarkably low adherence to a follow-up screening compared with their older women and those with more children counterparts, respectively. These interventions may include focused health education, peer support groups and individualised counselling aiming to improve their risk perception and health-seeking behaviour with regard to cervical cancer. Similar interventions should also be applied to WLHIV who receive normal screening outcomes during their first screening who also exhibited low adherence levels compared with their counterparts who had an abnormal screening. A structured and emotionally engaging health education should be provided to them emphasising that a normal screening outcome does not eliminate future risk.

## Data Availability

Data are available upon reasonable request. Data may be obtained from a third party and are not publicly available.
