# Supplementary material for: Adherence to follow-up cervical cancer screening and its predictors among women living with HIV in Moshi municipality, Tanzania: a hospital-based retrospective cohort study (2019–2024)
Source: BMJ Open. 2026 Sep 15;16(9):e113748. doi: 10.1136/bmjopen-2025-113748 (PMC13583725; doi:10.1136/bmjopen-2025-113748)
Supplement: Supplementary file 1 [file bmjopen-2025-113748.reviewer_comments.pdf]

PEER REVIEW HISTORY

BMJ Open publishes all reviews undertaken for accepted manuscripts. Reviewers are asked to complete a checklist review form and are provided with free text boxes to elaborate on their assessment. These free text comments are reproduced below.

ARTICLE DETAILS

Title (Provisional)

Adherence to Follow-up Cervical Cancer Screening and its Predictors among Women Living with HIV in Moshi Municipality, Tanzania: A Hospital-based Retrospective Cohort Study (2019–2024)

Authors

Doto, Dickson; Shirima, Laura J; Mmbaga, Blandina; Kinoko, Deogratius; Mboya, Innocent B; Ughh, Innocent H Peter; Mremi, Alex; Swai, Patricia; Mchome, Bariki

---

VERSION 1 - REVIEW

**Reviewer**	**1**
**Name**	**Ken-Amoah, Sebastian**
**Affiliation**	**University of Cape Coast, Department of Obstetrics and Gynaecology**
**Date**	**19-Jan-2026**
**COI**	**None**

Follow-up Cervical Cancer Screening among Women Living with HIV in Moshi Municipality, 2019–2024: Adherence and Predictors

General Comments

This manuscript addresses an important and policy-relevant question: adherence to follow-up cervical cancer screening among women living with HIV (WLHIV) in Tanzania. The focus on follow-up, rather than initial screening uptake, is appropriate and under-studied, particularly in low-resource settings. The study uses a large retrospective cohort derived from routine HMIS and HIV clinic data and the findings have potential implications for health programme design and health system strengthening.

However, in its current form, the manuscript requires major revision. Several methodological, conceptual, reporting and language issues undermine the strength and credibility of the manuscript. These concerns are largely surmountable through careful thorough revision.

Major Comments

1. Clarity and validity of the primary outcome (adherence)

The concept of “adherence to follow-up screening” is central to the manuscript, yet its use here lacks sufficient clarity and justification. Multiple adherence categories (early, good, poor, extremely poor) are reported, but the exact time thresholds defining these categories are not clearly described in the Methods. Additionally, it is not clear whether these categories are derived from national (Tanzanian) guidelines, WHO recommendations or author-defined cut-offs. Furthermore, the clinical and programmatic relevance of distinguishing between “poor” and “extremely poor” adherence is not adequately justified.

Threfore, I recommend that the authors should:

- explicitly define each adherence category in the Methods section, including exact time intervals.
- justify these cut-offs with reference to national or international guidelines, where possible.
- clarify how these categories inform clinical decision-making or programme monitoring.

2. Inconsistencies in study period and population
   - The title and several sections refer to 2019 - 2024, while the Methods describe inclusion of women first screened between 2019 and 2022.
   - It is not clear whether follow-up extended to 2024 for all participants or whether data availability varied.

I recommend that the authors harmonizes the study period across all sections of the manuscript and clearly describe the cohort entry period and the maximum follow-up duration.

3. Over-interpretation of associations

Although the study is observational and retrospective, parts of the Discussion imply causal mechanisms. Explanations related to provider behaviour, patient motivation and health system quality are speculative and not directly measured. Additionally, some interpretations are presented with undue certainty.

I recommend that the Discussion section should be revised to:

- use cautious language consistently
- clearly distinguish between observed associations and hypothesized mechanisms and
- emphasize the limitations of routine data for causal inference.

4. Recommendations not fully supported by findings

Some policy and clinical recommendations extend beyond what the data can support.

Examples:

- The recommendation to combine VIA, Pap smear and HPV DNA testing is not examined in the analysis and may not be feasible in the study context.
- Proposals to formally classify adherence categories at national level are premature without validation studies.

I recommend that the authors should align recommendations more closely with their findings and frame broader suggestions as areas for future research or policy discussion.

Minor Comments

1. Abstract

The abstract does not clearly state the study's added value. It does not also contain a clear definition of what constitutes "good adherence".

2. Introduction

The literature review could be more analytical and highlight why follow-up adherence is a critical gap distinct from screening uptake.

3. Methods

Authors should explicitly describe how missing data was handled and clearly explain data linkage procedures between HMIS and CTC2.

4. Results

Tables are dense and difficult to read; some could be moved to supplementary materials. Authors should ensure that all percentages and denominators are consistent.

5. Language and style

The manuscript contains many grammatical errors and awkward phrasing. A thorough language edit is recommended. The use of the word "Moreover" should be minimized. Transition between paragraphs should be improved.

**Reviewer**	**2**
**Name**	**Longatto Filho, Adhemar**
**Affiliation Institute**	**University of Minho, Life and Health Sciences Research**
**Date**	**21-Jan-2026**
**COI**	**None**

The authors evaluated one of the main limitations of screening programs in various parts of the world: adherence to the programs. In the case of women living with HIV, this issue is particularly alarming, since the development of serious HPV-induced lesions is more rapidly established. The study also addressed details of low adherence to programs by the female population and discussed elements related to this worrying situation. The work has many social merits associated with public health issues, which makes these data very relevant to the population studied. It is worthwhile to raise awareness among the health authorities responsible for investing in the improved performance of health systems in this area to avoid compromising the lives of many women.

VERSION 1 - AUTHOR RESPONSE

Thank you very much for your feedback on my manuscript I submitted for publication in your journal

I have worked on the comments you gave and now I am submitting the improved manuscript

you can find the new manuscript and the original manuscript which shows how comments were addressed

VERSION 2 - REVIEW

Reviewer 1

Name Ken-Amoah, Sebastian

Affiliation University of Cape Coast, Department of Obstetrics and Gynaecology

Date 15-Apr-2026

COI

Thank you for your revised submission. The rewritten Introduction section is more focused, the Methods section now describes data linkage and missing data procedures more explicitly and the removal of the unsupported multi-modal testing recommendation is appreciated. These improvements are acknowledged.

However, this second review has identified several concerns, some carried forward from the first round and several new, that must be addressed before the manuscript can be considered for acceptance.

Concerns requiring mandatory revision

1. Missing data-stated levels are inconsistent with Table 1

The Methods states that missingness in HIV-related variables was less than 10% and that no imputation was therefore performed. This is inconsistent with your own Table 1. Summing the reported percentages reveals approximately 30% missing for WHO HIV stage, 19% for viral load, and 27% for AHD status. These are not trivial levels. Please correct the stated missingness levels, conduct a sensitivity analysis comparing complete-case results with multiple imputation, and assess whether missingness is related to the outcome; non-adherent women may have less complete clinical records by virtue of reduced facility contact, which would represent informative rather than random missingness.

2. Odds ratios overestimate associations at 33% outcome prevalence

With outcome prevalence at 33.1%, logistic regression odds ratios systematically and substantially overestimate the magnitude of associations relative to risk ratios. The revised Methods invokes the 10% threshold as justification for retaining logistic regression, but this reasoning is inverted: the 10% rule recommends switching to Poisson regression with robust standard errors precisely when prevalence exceeds this level, because odds ratios increasingly diverge from risk ratios as prevalence rises. The AOR of 8.19 for abnormal screening outcome cannot be interpreted as an eight-fold increase in the probability of adherence. Please either re-run all primary analyses as Poisson regression with robust standard errors and report prevalence ratios or provide a formal mathematical justification for retaining odds ratios alongside an explicit statement that they overestimate the true effect sizes.

3. AHD finding based on critically sparse data

The AHD predictor is presented as a statistically significant finding and interpreted as clinically meaningful. However, only five participants with AHD adhered to follow-up. An odds ratio derived from five events in one cell is highly unstable, and the confidence interval of 0.09–0.66 reflects this uncertainty. Please flag this finding explicitly as exploratory in the Results, add a statement of caution in the Discussion and avoid building policy recommendations around it pending replication in a larger sample.

4. 'Early adherence' category requires methodological justification

Among the 1,019 women who adhered, 291 (28.6%) demonstrated early adherence, defined as attending before the scheduled date. This is a striking finding that the manuscript does not explain. In Tanzanian cervical cancer screening delivery, early attendance may reflect opportunistic presentation during routine CTC or ANC visits rather than scheduled adherence behaviour; these are not behaviourally equivalent. Including early attendees in the adherence numerator without justification inflates the headline adherence rate and may confound the predictor analysis. Please provide a clinical or programmatic rationale for treating early attendance as adherence or conduct a sensitivity analysis excluding early attendees from the primary outcome and report whether the predictor findings change.

5. Differential follow-up time by cohort entry year

Women recruited in 2022 had a maximum of approximately two years of observation at data closure, while those recruited in 2019 had up to five. This heterogeneity directly affects the validity of the extremely poor adherence category and may have inflated apparent non-adherence in later-recruited participants. Please either: (a) report adherence rates stratified by cohort entry year to assess whether this affects findings; or (b) limit the primary analysis to women with a minimum of two years of observation and present the full cohort as a sensitivity analysis.

6. Over-interpretation of associations in the Discussion

The Discussion continues to assert mechanistic explanations (provider behaviour, patient motivation, facility tracking system quality, health awareness) for associations where none of these variables were measured. While contextual interpretation is appropriate, the Discussion does not contain a statement acknowledging that this retrospective analysis of routine registry data cannot establish causal pathways. Please add an explicit acknowledgement of the key unmeasured confounders relevant to this setting: transport access and distance to facility, health literacy, HIV stigma, socioeconomic status, and facility-level staffing and recall infrastructure. The conclusion attributing superior adherence in private facilities to better systems quality also requires qualification: in this dataset, private facilities are a Catholic mission hospital and an NGO reproductive health clinic serving populations who have actively sought out non-governmental services. This self-selection means the observed difference likely reflects patient case-mix as much as facility quality, and the Discussion should say so.

Additional corrections required

7. Abstract confidence interval error

The abstract reports the parity AOR confidence interval as "95% 1.21–4.84," which appears to be a copy error from the age group AOR. The correct CI per Table 3 is 1.11–2.49. Please correct.

10. Reference errors

References 15 and 16 cited for the conceptual framework appear to be a Malaysian recall-reminder trial and a Portuguese screening protocol. Neither provides a theoretical basis for adherence behaviour among WLHIV. Please identify and cite appropriate sources. References 2 and 4 appear to be duplicate citations of the same Sedani et al. paper and should be resolved.

8. Table 2 denominator direction

The revised Table 2 presents within-group column percentages rather than the row percentages used in the original. This change is not signalled and makes clinical interpretation more difficult. Please state the direction of percentages and the denominator basis clearly in the table footnote.

9. Language

Grammatical errors persist, including "the findings have showed", “undertake”(instead of 'undertook') and "those who were had their first screening." The word "Moreover" is used more than ten times in the Discussion. Pay attention to punctuation. A professional language edit is required before resubmission.

The study addresses an important and under-researched question in East African cervical cancer prevention and the dataset is a genuine strength. These concerns are surmountable and the manuscript has real potential for publication once they are resolved.

VERSION 2 - AUTHOR RESPONSE

HIGHLIGHTS OF THE KEY ISSUES ADDRESSED IN THE REVISED MANUSCRIPT VERSION

In the Abstract section: Minor changes have been made as per changes in the methodology, results, discussion and conclusion.

Introduction section: No changes were made

In the Methodology section: To address the concern regarding "differential follow-up time by cohort entry year," I acknowledged that WLHIV recruited in different years (2019, 2020, 2021, and 2022) had varying durations of follow-up. Based on the operational definition of adherence categories used in the study, it was truly difficult to justify the classification of "extremely poor adherence" and "non-adherence" among WLHIV recruited in 2022, as these categories required at least two complete follow-up years after the scheduled follow-up date to be adequately assessed. This cannot be the case for WLHIV recruited in 2022. Consequently, WLHIV recruited in 2022 were excluded from the analysis, and only those recruited between 2019 and 2021 were retained, as all had a minimum of two years of follow-up, consistent with the reviewer's recommendation. This significantly changed the number of study's participants.

Furthermore, although the retained cohorts still differed in their year of recruitment, the challenge of classifying extremely poor adherence was eliminated. This is because any follow-up screening attended more than one year after the scheduled date could be consistently classified as extremely poor adherence, regardless of the extent of the additional delay beyond that period.

Regarding the handling of missing data, a sensitivity analysis was conducted to assess whether the analysis including variables with missing values—namely AHD development status, HIV viral load, and WHO HIV stage—differed from the analysis in which the missing values for these variables were addressed using mode imputation. Significant differences were observed between the two analyses. Consequently, it was concluded that the variables with missing values addressed through mode imputation would be retained and used in the multivariable model. In addition, further justification for the classification of early adherence to follow-up screening was provided in the revised manuscript. Furthermore, Poisson regression with robust standard errors was used to determine the association between adherence to follow-up screening and its predictors, replacing the previously used logistic regression model.

In the results section: Row presentation of the Chi-square table has been restored as recommended by the reviewer. Other changes are per the changes in the methodology pointed above. Also, a caution on interpreting the results for AHD development status has been added. In addition, an improved way (justification) for the findings about WLHIV who undertook their first screening in private healthcare facilities being more likely to adhere to a follow-up screening as compared to those who went to public healthcare facilities has been provided. I invite you to read the citations given for much deeper understanding, as this finding could be a result of the deeply rooted disparity in the quality of services (not technical capacity) between public and private healthcare centres in Tanzania and other similar settings in SSA.

In the discussion section: The mechanistic explanations previously provided such as provider behaviour, patient motivation, quality of facility tracking systems, and health awareness to

justify the observed adherence patterns to follow-up screening—despite these variables not being directly measured in the study—were revised and replaced with more cautious, realistic, and evidence-based interpretations consistent with the available study findings.

In the Conclusion section: Minor changes were made to reflect the changes in the methodology and discussion sections.

In the reference section: The referencing issue identified in references 15 and 16 related to the conceptual framework was addressed by replacing them with more appropriate and relevant reference.

Language issue: All grammatical errors pointed out has been addressed in the new manuscript

Reviewer 1

Comment 1: Missing data-stated levels are inconsistent with Table 1

The Methods states that missingness in HIV-related variables was less than 10% and that no imputation was therefore performed. This is inconsistent with your own Table 1. Summing the reported percentages reveals approximately 30% missing for WHO HIV stage, 19% for viral load, and 27% for AHD status. These are not trivial levels. Please correct the stated missingness levels, conduct a sensitivity analysis comparing complete-case results with multiple imputation, and assess whether missingness is related to the outcome; non-adherent women may have less complete clinical records by virtue of reduced facility contact, which would represent informative rather than random missingness.

Author's response: After carefully data checks it was revealed that there was indeed a serious level of missingness in some variables. Therefore, for variables with substantial levels of missing data, namely **AHD development status, HIV viral load, and WHO HIV stage**, a sensitivity analysis was conducted to assess the impact of missing values.

The results indicated that including these variables after handling the missing data using **mode imputation** in the multivariable analysis provided more reliable and informative analyses than using these variables in their original form (with missing values). Therefore, the imputed variables were retained in the final analysis. This approach was also consistent with the reviewer's recommendation.

These changes have been addressed in 14th-17th lines of page 6 (in the section titled *"statistical analysis"*).

Reviewer 1

Comment 2: Odds ratios overestimate associations at 33% outcome prevalence

With outcome prevalence at 33.1%, logistic regression odds ratios systematically and substantially overestimate the magnitude of associations relative to risk ratios. The revised Methods invokes the 10% threshold as justification for retaining logistic regression, but this reasoning is inverted: the 10% rule recommends switching to Poisson regression with robust standard errors precisely when prevalence exceeds this level, because odds ratios increasingly diverge from risk ratios as prevalence rises. The AOR of 8.19 for abnormal screening outcome cannot be interpreted as an eight-fold increase in the probability of adherence. Please either re-run all primary analyses as Poisson regression with robust standard errors and report prevalence ratios or provide a formal mathematical justification for retaining odds ratios alongside an explicit statement that they overestimate the true effect sizes.

Author's response: It's true that the use of odds ratio (logistic regression) can potentially overestimate the association of dependent variable and independent variables in this study primarily due to the study design used (cohort study design).

In order to address this, a Poisson regression model with robust standard errors has replaced logistic regression as the model for multivariable analysis in the revised manuscript. This is in line with the reviewer recommendation.

1. These changes have been addressed in the 8th line of page 6 in the revised manuscript (in the section titled discuss statistical analysis).

2. These changes have been addressed in the 12nd line of page 6 in the revised manuscript (in the section titled discuss statistical analysis).

3. In order to address this, a Poisson regression model with robust standard errors (prevalence ratio) has replaced logistic regression (odds ratio) as the model for multivariable analysis in the revised manuscript. This is in line with the reviewer recommendation.

As a results, significant changes have been observed in the results of the multivariable analysis in almost every single variable. These changes can be seen in 1-12nd lines of the only paragraph in page 10 and the first two lines of page 11 of the revised manuscript.

4. As a results, significant changes have been observed in the results of the multivariable analysis **in table 3** in almost every single variable as compared to when logistic regression (odds ratio) was used in the previous manuscript. These changes can be seen in pages 11 and 12 (in table 3) in the revised manuscript.

Reviewer 1

Comment 3: AHD finding based on critically sparse data

The AHD predictor is presented as a statistically significant finding and interpreted as

clinically meaningful. However, only five participants with AHD adhered to follow-up. An odds ratio derived from five events in one cell is highly unstable, and the confidence interval of 0.09–0.66 reflects this uncertainty. Please flag this finding explicitly as exploratory in the Results, add a statement of caution in the Discussion and avoid building policy recommendations around it pending replication in a larger sample.

Author's response: I agree with the reviewer observation that the results from this part should be interpreted cautiously even though they are statistically significant. So, in line with the reviewer's recommendation, a cautionary statement has been included to indicate that the results from this part should not be taken without further considerations and should not be used to make policies, until confirmed/replicated in similar studies from which number of WLHIV who had developed AHD is sufficient

These changes have been addressed in the 16th-30th lines in page 15 in the revised manuscript.

Reviewer 1

Comment 4: 'Early adherence' category requires methodological justification
Among the 1,019 women who adhered, 291 (28.6%) demonstrated early adherence, defined as attending before the scheduled date. This is a striking finding that the manuscript does not explain. In Tanzanian cervical cancer screening delivery, early attendance may reflect opportunistic presentation during routine CTC or ANC visits rather than scheduled adherence behaviour; these are not behaviourally equivalent. Including early attendees in the adherence numerator without justification inflates the headline adherence rate and may confound the predictor analysis. Please provide a clinical or programmatic rationale for treating early attendance as adherence or conduct a sensitivity analysis excluding early attendees from the primary outcome and report whether the predictor findings change.

Author's response: The decision to retain this subcategory is based on observations made during data acquisition stage, **where a considerable number of WLHIV returned for follow-up screening earlier than their scheduled appointment dates**. This observation raised important practical considerations regarding whether these clients were fully aware of their correct follow-up schedules or whether some early returns were influenced by other factors, such as bad post-screening experiences such as side effects following treatment of precancerous lesions among those with abnormal first screening outcomes, including symptoms like bleeding and related discomfort.

Keeping this group separate is therefore important because it reflects a real and distinct pattern in follow-up behavior. Recognizing this pattern may also help improve counselling and strengthen program guidance on the appropriate timing for follow-up screening in routine care. It also creates a platform or justification for other researchers to further

investigates what could it mean and its implications in the cervical cancer screening initiative in the country and other similar settings.

The discussion of this matter can be seen in the 4th-5th lines of the only paragraph of page 8 in the revised manuscript.

Reviewer 1

Comment 5: Differential follow-up time by cohort entry year
Women recruited in 2022 had a maximum of approximately two years of observation at data closure, while those recruited in 2019 had up to five. This heterogeneity directly affects the validity of the extremely poor adherence category and may have inflated apparent non-adherence in later-recruited participants. Please either: (a) report adherence rates stratified by cohort entry year to assess whether this affects findings; or (b) limit the primary analysis to women with a minimum of two years of observation and present the full cohort as a sensitivity analysis.

Author's response

In order to address this, I have made changes in the recruitment period aiming to exclude participants recruited in 2022 in the revised manuscript.

Therefore, the recruitment period changed from 2019–2022 to 2019–2021. This ensured that all the remained study participants had at least two years of follow-up after their scheduled follow-up date, in accordance with the reviewer's recommendation.

This aim to better substantiate extremely poor adherence subcategory and non-adherent WLHIV, something which could not been done for participants recruited in 2022, simply because they could not be followed for complete 2 years after their supposedly date for follow-up screening. Also, it is acknowledged that WLHIV recruited in earlier years (2019 and 2020) had a longer overall follow-up period than those recruited in 2021. However, this does not affect the classification of adherence because all retained participants had at least two years of follow-up after their scheduled follow-up date, which was sufficient to ascertain all adherence categories, including extremely poor adherence and non-adherence. Adherence classification was based on predefined time intervals relative to the scheduled follow-up date rather than the total duration of follow-up or year of recruitment.

Therefore, all participants were assessed using the same criteria regardless of when they entered the cohort. For example, a participant who attended screening more than one year after the scheduled follow-up date was classified as having extremely poor adherence, irrespective of her recruitment year. Consequently, differences in overall follow-up duration among the retained cohorts did not influence adherence categorization or the interpretation of study findings.

Consequently, the total number of recruited participants was changed i.e. from 3,125 to 2,066. This change also affected the number of participants excluded due to exclusion criteria, as well as the final number of participants retained in the study.

1. These changes have been addressed in the 1st, 2nd and 3rd lines of the 4th paragraph in page 4 and 2nd line of the 1st paragraph of page 5. These pages belong to the section titled *"study participants section*".

2. So, because of that, the numbers in the characteristics of study participants changed significantly as it can be observed in this section. These changes can be seen in 9th-19th lines of page 7 (in the results- participants characteristics section) in the revised manuscript.

3. Consequently, the total number of recruited participants was changed i.e. from 3,125 to 2,066. As a results, the numbers in the participants characteristics have also significantly changed as it can been seen in the Table 1 in the revised manuscript in page 7-8.

4**.** To address the reviewer's concern, the recruitment period was revised from 2019–2022 to 2019–2021, thereby excluding participants recruited in 2022 from the analysis. This ensured that all included participants had a minimum of two years of follow-up after their scheduled follow-up date, in line with the reviewer's recommendation.

As a result of this revision, the total number of study participants decreased from 3,125 to 2,066. Consequently, the number of participants who adhered to follow-up screening, as well as the distribution across adherence subcategories (early adherence, good adherence, poor adherence, and extremely poor adherence), changed substantially. These changes have been reflected throughout the revised manuscript.

Reviewer 1

Comment 6: Over-interpretation of associations in the Discussion
The Discussion continues to assert mechanistic explanations (provider behaviour, patient motivation, facility tracking system quality, health awareness) for associations where none of these variables were measured. While contextual interpretation is appropriate, the Discussion does not contain a statement acknowledging that this retrospective analysis of routine registry data cannot establish causal pathways. Please add an explicit acknowledgement of the key unmeasured confounders relevant to this setting: transport access and distance to facility, health literacy, HIV stigma, socioeconomic status, and facility-level staffing and recall infrastructure. The conclusion attributing superior adherence in private facilities to better systems quality also requires qualification: in this dataset, private facilities are a Catholic mission hospital and an NGO reproductive health clinic serving populations who have actively sought out non-governmental services. This self-selection means the observed difference likely reflects patient case-mix as much as facility quality, and the Discussion should say so.
Additional corrections required

1. Author's comment: The first paragraph of the discussion section has now been revised to make the interpretation of findings more cautious. Specifically, any explanations that previously implied causal or pathway relationships have been replaced with more balanced and appropriate interpretations consistent with the study design.

In addition, in response to the reviewer's recommendation, the discussion now acknowledges important unmeasured factors that could have influenced the findings (low adherence) in this study. These include access to transport and distance to healthcare facilities, levels of health literacy, HIV-related stigma, socioeconomic conditions, as well as facility-level factors such as staffing levels and the availability of patient recall systems. These changes have been addressed in the 1st – 10th lines of the first paragraph of page 12 in the revised manuscript.

2. Author's response: In response to this comment, a more polished explanation has been provided in the second paragraph of the discussion section, whereby earlier non-causal pathway inferences appeared in the previous manuscript have been revised and replaced with interpretations that are more aligned with the study design in the revised manuscript. Also, the language/grammar in that particular paragraph has also been improved to improve clarity and meaning. All of these are in line with the reviewer's recommendations.

These changes have been addressed in 1st-8th lines in page 12, and in 9th-19th lines in page 13 (in discussion section).

3. **Author's response**: In response to this comment, a more polished explanation has been provided in the second paragraph of the discussion section, whereby earlier non-causal pathway inferences appeared in the previous manuscript have been revised and replaced with interpretations that are more aligned with the study design in the revised manuscript. Also, the language/grammar in that particular paragraph has also been improved to improve clarity and meaning. All of these are in line with the reviewer's recommendations.

These changes have been addressed in 1st-8th lines in page 12, and in 9th-19th lines in page 13 (in discussion section).

4. Author's response: In addressing the reviewer's comment on over-interpretation of associations in the conclusion attributing superior adherence in private facilities compared to public facilities, **the first step** has been to rephase the previous given explanations and adding some new insight into it

Why so: In the Tanzanian context and other settings in sub-Saharan Africa, it is a well-documented fact that clients attending private healthcare facilities often report higher levels of satisfaction, largely due to perceived better service delivery and stronger provider–client relationships, rather than necessarily superior clinical or technical capacity. Although it may sometimes be more costly to get clinical services in private facilities as compared to public ones, it may be worth it for many clients because of the stated reasons. This may therefore partly explain why WLHIV who received their first screening in private facilities were more likely to return for follow-up screening compared to those attending public facilities.

In contrast, public healthcare facilities often face challenges such as understaffing, which can lead to long waiting times, congestion and queues in the healthcare facility particularly for RMNCAH services (which include cervical cancer screening) which typically has a lot of clients on the daily basis. These experiences may be one of the reasons to discourage many WLHIV who had their first screening in public facilities from returning to a follow-up care.

Moreover, these interpretations are consistent with existing literatures and other supporting references from our setting (Tanzania and SSA), and you can find their link below for reference.

1. DOI:10.61538/pajbm.v7i1.1293

2. DOI: 10.1186/1472-6963-11-203

3. chrome-extension://efaidnbmnnnibpcajpcglclefindmkaj/https://www.repoa.or.tz/wp-content/uploads/2021/02/SDI-Health-Designed-Sept-2020.pdf

These changes have been addressed in 12nd – 29th lines in page 13 in the new manuscript.

Reviewer 1

Comment 8: Table 2 denominator direction

The revised Table 2 presents within-group column percentages rather than the row percentages used in the original. This change is not signalled and makes clinical interpretation more difficult. Please state the direction of percentages and the denominator basis clearly in the table footnote.

---

1. Author's response: In order to address this in the revised manuscript, the chi-square test results have been presented by rows rather than by columns, as was the case in the previous version. This format has been restored to improve readability and facilitate interpretation of the findings. The revision was made in accordance with the reviewer's recommendation.

These changes have been addressed in 1st-12nd lines in the only paragraph of page 9 in the revised manuscript.

---

2. Author's response: In order to address this in the revised manuscript, the results of the chi-square test table have been presented by rows rather than by columns, as was the case in the previous version. This format has been restored to improve readability and facilitate interpretation of the findings. The revision was made in accordance with the reviewer's recommendation. These changes have been addressed in 1st-12nd lines in the only paragraph of page 9 in the revised manuscript.

Reviewer 1

Comment 9: Language

Grammatical errors persist, including "the findings have showed", “undertake” (instead of 'undertook') and "those who were had their first screening." The word "Moreover" is used more than ten times in the Discussion. Pay attention to punctuation. A professional language edit is required before resubmission.

The study addresses an important and under-researched question in East African cervical cancer prevention and the dataset is a genuine strength. These concerns are surmountable and the manuscript has real potential for publication once they are resolved.

1. Author’s response: I have revised the word *“undertaken”* to read “undertook” to improve the grammatical meaning as suggested by the reviewer 1

These changes have been addressed in the first line of the 4th paragraph in page 4 of the revised manuscript.

2. Author’s response: To improve the language, grammar, clarity, and overall flow of the manuscript, the paragraph marked with track changes has been replaced with the revised version below. The new paragraph is more concise, clearer, and better conveys the intended meaning.

These changes have been addressed in 30th-31st lines of page 6 and 1st-8th lines of page 7 in the revised manuscript.

3. **Author’s response:** a part of the sentence which reads *“…. the findings have showed that older WLHIV were more likely to ……”* has been revised to read “…. the findings have shown that older WLHIV were more likely to ….”

These changes can be found in the 10th line of page 14 in the revised manuscript.

4. **Author’s comment:** In line with the reviewer’s comment to improve the grammar and meaning of the sentences in the revised manuscript, I have made language revision in the whole second paragraph of the conclusion part, whereby its rich in content, meaning and more importantly grammatically correct.

These changes can be seen in the entire 2nd paragraph of the conclusion section (in 8th -19th lines of page 16).

5. **Author’s response:** a part of the sentence which reads *“…. the findings have showed that older WLHIV were more likely to ……”* has been revised to read “…. the findings have shown that older WLHIV were more likely to ….”

These changes can be found in the 10th line of page 14 in the revised manuscript.

Reviewer 1

Comment 10. Reference errors
References 15 and 16 cited for the conceptual framework appear to be a Malaysian recall-reminder trial and a Portuguese screening protocol. Neither provides a theoretical basis for adherence behaviour among WLHIV. Please identify and cite appropriate sources. References 2 and 4 appear to be duplicate citations of the same Sedani et al. paper and should be resolved.

Author's response: In addressing this, the new reference study has replaced the previous ones as its findings are more in line with our conceptual framework.

I invite the reviewer to read it (reference 35) to see that alignment.

This changes can be seen in figure 3 in the separate document sent along side this document

---

VERSION 3 - REVIEW

**Reviewer**	**1**
**Name**	**Ken-Amoah, Sebastian**
**Affiliation**	**University of Cape Coast, Department of Obstetrics and Gynaecology**
**Date**	**07-Jul-2026**
**COI**	

Thank you for your careful and constructive responses to the previous review. The manuscript has improved substantially, and the revisions have addressed the major methodological and interpretive concerns raised during the initial review. In particular, the changes to the analytical approach, clarification of the study period, improved presentation of the results and more balanced interpretation of the findings have strengthened the manuscript considerably.

Before the manuscript is ready for publication, I recommend that the authors address the following minor issues:

1. **Missing data:** Please provide a clearer and more concise description of the extent of missing data and the approach used to handle it, ensuring consistency between the Methods and Results sections.
2. **Language:** A final careful proofread is recommended to correct the remaining grammatical and typographical errors and improve the overall flow of the manuscript.
3. **References:** Please carefully review the reference list to ensure consistency in formatting and remove any duplicate or incorrect citations.

Subject to these minor revisions, I believe the manuscript will be suitable for publication. I commend the authors for their thorough revision and thoughtful engagement with the reviewer comments.

VERSION 3 - AUTHOR RESPONSE

Reviewer 1: Comment 1

Missing data: Please provide a clearer and more concise description of the extent of missing data and the approach used to handle it, ensuring consistency between the Methods and Results sections.

---

1. Author's response: In response to this comment, the paragraph describing the extent of missing data and the approach used to address it in the previous version of the manuscript has been revised and replaced with a more comprehensive and robust description. The revised text is presented below and has been edited for clarity, conciseness, and grammatical accuracy to enhance readability and ensure consistency between the Methods and Results sections of the manuscript.

These revisions can be found in the last paragraph of page 6 and continue through the first two lines of page 7 of the revised manuscript.

2. **Author's response:** In response to this comment, a grammatical correction was made whereby "are" (plural) was replaced with "is" (singular) to ensure subject–verb agreement. This revision is consistent with the reviewer's recommendation to address grammatical errors throughout the manuscript

These changes can be seen in 9th line of the second paragraph in page 14

Reviewer 1: Comment 2

Language: A final careful proofread is recommended to correct the remaining grammatical and typographical errors and improve the overall flow of the manuscript.

---

1. Author's response: To address this comment, the word "had" has been removed in the revised manuscript to enhance grammatical correctness. This is in line with the reviewer's remarks.

This change can be seen in first line of the methodology part of the abstract section in page 2

2. Author's response: In addressing this comment, this part of the paragraph has been revised to ensure consistency in verb tense by changing the relevant statements to the past tense.

These changes can be seen in the last paragraph of the results part of the Abstract section in page 2

3. **Author's response:** In addressing this comment, several words have been replaced with more appropriate and meaningful terms to improve clarity. Additionally, the sentence structure and flow in this section have been revised to enhance grammatical correctness and readability.

These changes can be seen in the conclusion of the abstract in Chapter 2 of the manuscript.

4. Author's response: In addressing this, a word "Moreover" has been removed from the sentence as it appeared too much in the text. This is in line with the reviewer's recommendations**.**

These changes can be seen in the 6th line of the first paragraph of the introduction section in page 3

5. Author's response: In addressing this, a word "Also" has been removed from the sentence as it appeared too much in the text. This is in line with the reviewer's recommendations**.**

These changes can be seen in the 3rd line of the second paragraph of the introduction section in page 3

6. Author's response: In addressing this, a word "However" has been replaced with the words "To date" as it has appeared too much in the text. This is just to improve the overall text flow. This is in line with the reviewer's recommendations**.**

These changes can be seen in the 2nd line of the third paragraph of the introduction section in page 4

7. Author's response: In addressing this, VIA has been written alongside its long form (it has been defined) at its first appearance in the text. This aims to improve readability and overall text flow in line with the reviewer's recommendations.

These changes can be seen in the 6th - 7th lines of the fourth paragraph of the methodology section in page 5

8. Author's response: In addressing this, the highlighted part of the paragraph has been revised and rewritten to improve clarity and overall text flow.

These changes can be seen in the 08th – 14th lines of the second paragraph of the statistical analysis section in page 6 of the revised manuscript (main document).

9. Author's response: To address this comment, the first four lines of the paragraph have been revised and replaced with a more concise and coherent version to enhance grammatical correctness, readability, and the overall quality of the revised manuscript.

These revisions can be found in lines 1–8 of the second paragraph on page 7 of the revised manuscript.

10. Author's response: To address this comment, a part of the sentence "…. It was observed that..." has been replaced with words "… we found that …".

This change aimed to use active voice in this regard which is add more weight into the message being communicated. It also increases readability, and the overall quality of the revised manuscript.

These revisions can be found in 1st-2nd lines of the first paragraph of page 11 in the revised manuscript.

11. **Author's response:** In response to this comment, correction on this grammatical error, was done by replacing the word "they" with "this" . This is in line with the reviewer's remarks.

These changes can be seen in 7th line of the second paragraph of page 12

Reviewer 1: Comment 3

References: Please carefully review the reference list to ensure consistency in formatting and remove any duplicate or incorrect citations.

---

1. To address this comment, Reference 11 has been revised to improve its formatting and readability in accordance with the journal's citation guidelines. It should also be noted that this reference is not a published journal article, but rather a national strategic plan that is publicly available online.

These changes can be seen in page 19 of the revised manuscript (reference 11 in the reference section)

2. To address this comment, Reference 22 has been revised to remove the quotation marks at the start and end of it in accordance with the journal's citation guidelines. It should also be noted that this reference is an organization's survey report rather than a traditional peer-reviewed journal article.

These changes can be seen in page 19 of the revised manuscript (reference 19 in the reference section)

3. To address this, Reference 29 has been removed from the list of references as it's a duplicate of reference 14.

These changes can be seen in page 18 and 19 in the main document (in the list of reference)

4. To address this, Reference 33 has been removed from the list of references as it's a duplicate of reference 7

These changes can be seen in page 18 and 19 in the main document (in the list of reference)
